# Comparison of the digestive efficiency of extruded diets fed to ferrets (*Mustela putorius furo)*, dogs (*Canis familiaris*) and cats (*Felis catus*)*

**DOI:** 10.1017/jns.2014.30

**Published:** 2014-09-25

**Authors:** Fabiano C. Sá, Flavio L. Silva, Márcia de O. S. Gomes, Márcio A. Brunetto, Rodrigo S. Bazolli, Thiago Giraldi, Aulus C. Carciofi

**Affiliations:** 1 College of Agrarian and Veterinarian Sciences (FCAV), São Paulo State University (UNESP), Via de Acesso Professor Paulo Donato Castellane, Jaboticabal 14·884-900, SP, Brazil; 2 Camilo Castelo Branco University (UNICASTELO), Av. Hilário da Silva Passos, 950, Descalvado 13 690-000 SP, Brazil; 3 Faculty of Veterinary Medicine and Animal Science (FMVZ), University of São Paulo (USP), Av. Duque de Caxias Norte, 225, Pirassununga 13·635-900 SP, Brazil

**Keywords:** Canine nutrition, Digestibility, Feline nutrition, Mustelidae, ATTD, apparent total tract digestibility, BW, body weight, CF, crude fibre, CP, crude protein, HC, high carbohydrate, LC, low carbohydrate, MC, moderate carbohydrate, NFE, nitrogen-free extract

## Abstract

The digestive tract of ferrets is anatomically simple, with no caecum, ileocolic valve or external differentiation between the transition of ileum and colon. The species has a short large intestine that provides minor contributions to the digestive process. Aiming to better understand the digestibility efficiency of ferrets, the present study compared the digestibility of extruded diets with different amounts of macronutrients fed to dogs, cats and ferrets. Three formulations for cat maintenance were used (values in % of DM basis): high carbohydrate (HC; nitrogen-free extract (NFE) = 54 %, protein = 31 % and fat = 8 %); moderate carbohydrate (MC; NFE = 37 %, protein = 41 % and fat = 10 %); and low carbohydrate (LC; NFE = 19 %, protein = 46 % and fat = 23 %). Apparent total tract macronutrient digestibility was determined by the method of total collection of faeces. Results were compared by ANOVA, considering the diet and species effects and their interactions. Means were compared by the Tukey's test (*P* < 0·05). Dogs and cats presented similar food intakes, but ferrets consumed almost two times more food (g/kg body weight). Species × diet interactions were verified for apparent total tract digestibility (ATTD; *P* < 0·05). Ferrets presented lower DM digestibility than dogs and cats for all three diets (*P* < 0·05), lower NFE digestibility than dogs for the three diets and lower NFE digestibility than cats for the HC and LC diets (*P* < 0·05). For crude protein (CP), ferrets presented lower ATTD than dogs and cats (*P* < 0·05), whereas for fat, dogs and ferrets presented similar ATTD, and higher values than those presented by cats (*P* < 0·05). Kibble diets had a lower DM, CP and NFE digestibility when fed to ferrets compared with dogs and cats. Fat digestibility was similar between dogs and ferrets and higher than that for cats.

Ferrets are mammals of the order Carnivora, having a digestive tract that is anatomically simple, with no caecum, ileocolic valve or external differentiation between the transition of ileum and colon. In addition, the species has a short colon length that provides minor contributions to the digestive process. These anatomic features result in a fast transit time of food from ingestion to excretion, that can last from 1 to 3 h^(^^1^^)^. Although being a popular companion species, only a few studies assessing the digestive efficiency of ferrets have been found in the literature. In mink (*Mustela vison*), another member of the *Mustelidae* family, fat digestibility can vary depending on its nature, source and amount in the diet^(^^2^^)^. Comparing the digestibility of a food fed to a canid species, the blue fox (*Alopex lapogus*), and that of the mink, higher carbohydrates and protein digestibility was reported in blue fox^(^^3^^)^. Those authors also reported that increased carbohydrate concentrations resulted in decreased digestibility of protein and fat for mink that was not observed for the blue fox, which tolerated higher concentrations of starch^(^^3^^)^. In a recent publication^(^^4^^)^, data on the digestibility coefficients available from the literature were collected to assess whether differences in protein and fat digestibility could be detected between groups of wild carnivores (canids, felids, hyenids, mustelids, pinnipeds and ursids) and domestic carnivores. Those authors observed that mustelids have patterns of protein digestion that are similar to both dogs and cats, and patterns of fat digestion that are similar to dogs but not cats.

Among the domesticated animals of the Carnivora, domestic dogs and cats have been the most studied. Several books and scientific papers have been published characterising their digestive processes^(^^5^^,^^6^^)^. To add information about the adequate feeding of ferrets, the present study compared the digestibility of this mustelid with one canid, the domestic dog, and one felid, the domestic cat, using for this purpose three extruded diets with different macronutrient compositions.

## Material and methods

The assay was conducted at the Laboratory of Research on Nutrition and Nutritional Diseases of Dogs and Cats, UNESP, Jaboticabal, Brazil. All procedures were approved by the Ethics and Animal Welfare Committee of the College of Agrarian and Veterinarian Sciences, São Paulo State University.

### Animals, diets and study design

The experiment was conducted with eighteen ferrets (1·5 ± 0·0 years old and 1·2 ± 0·2 kg body weight (BW)), eighteen dogs (3·2 ± 1·2 years old and 11·0 ± 1·2 kg BW) and eighteen cats (4·1 ± 0·8 years old and 4·5 ± 0·9 kg BW). The health of all animals was confirmed prior to the start of the study.

Three extruded kibble diets with different macronutrient compositions were used (analysed values, on DM basis): high carbohydrate (HC; 54 % nitrogen-free extract (NFE), 31 % crude protein (CP), 8 % fat, 2·2 % crude fibre (CF) and 5·3 % ash); moderate carbohydrate (MC; 37 % NFE, 41 % CP, 10 % fat, 3·4 % CF and 8 % ash); and low carbohydrate (LC; 19 % NFE, 46 % CP, 23 % fat, 3·8 % CF and 8·9 % ash). Diets were formulated according to the European Pet Food Industry Federation^(^^7^^)^ recommendations for cat maintenance. The base of the diets comprised broken rice, maize, poultry by-product meal, maize gluten, poultry fat and beet pulp. The LC diets also included isolated soya protein. Diets were produced in commercial pet food plants (HC and MC diets on Mogiana Alimentos S.A. and LC diet on Dog Star Foods).

Dogs and cats were fed calculated amounts of food that were determined by calculating the energy required for weight maintenance for each animal and the metabolisable energy of the food estimated according to the National Research Council^(^^6^^)^. Ferrets were fed *ad libitum*. Food not consumed was collected and weighed, and food intake was calculated during all experimental periods. Water was provided *ad libitum*.

Animals were housed individually in stainless steel metabolic cages (1·0 × 1·0 × 1·0 m^3^ for dogs and 0·8 × 0·8 × 0·9 m^3^ for cats and ferrets). The digestibility test was performed through the total collection of the faeces method, with 10 d of adaptation, and 5 d of total collection of faeces for dogs and 10 d of total faeces collection for cats and ferrets. The longer collection periods for cats and ferrets were required to collect enough faeces for the laboratory analysis. Faeces were collected twice a day, weighed, pooled by animal and frozen at −20°C.

The experiment was organised in a 3 × 3 factorial arrangement of treatments, with three species (ferret, dog and cat) and three diets (LC, MC and HC) to yield nine experimental treatments. It was conducted in a randomised design, with eighteen animals of each species and six repetitions (animals) per treatment.

### Chemical analysis and calculations

At the end of the collection period, faeces were thawed, homogenised and pooled by animal. Before performing laboratory tests, faeces were dried in a forced-air oven at 55°C for 72 h (Fanem) and ground in a cutting mill (MOD 340, ART LAB) with a 1 mm sieve. Organic matter was calculated as 100 – ash and NFE were obtained by the difference of DM and the sum of CP, acid-hydrolysed fat, CF and ash. Diets and faeces samples were analysed for DM (method 934.01), CP (method 954.01), CF (method 962.09), ash (method 942.05) and acid-hydrolysed fat (method 954.02) according to the standard methods of the Association of American Feed Control Officials^(^^8^^)^. All analyses were carried out in duplicate and repeated when the CV was greater than 5 %. Nutrient digestibility was calculated according to the method of Merchen^(^^9^^)^.

### Statistical analysis

The experiment followed a 3 × 3 factorial arrangement (three species and three diets), with nine treatments and six repetitions per diet. Data were analysed in a completely randomised design using the General Linear Model procedures of the Statistical Analysis Systems statistical software package version 9.0 (SAS Institute, Cary, NC, USA). The experimental unit was one animal. The model sums of squares were separated into diet and species effects, and their interactions. The interactions among the variables tested (diet and animal species) were analysed with the SLICE statement. When significant differences were detected in the *F* test of ANOVA, multiple comparisons of the means were performed using the Tukey's test. Values of *P* < 0·05 were considered significant. All data complied with the assumptions of ANOVA models.

## Results

No animal refused the food and all diets were consumed. The animals maintained constant BW during the study (data not shown). Faeces production was adequate, with no episodes of diarrhoea. For nutrient intake, diet × species interactions were found (*P* < 0·05). Ferrets presented higher nutrient intake for the three diets (g/kg BW per d) than dogs and cats (*P* < 0·01), consuming approximately two times more food to maintain constant BW (Table 1). Within each species, DM consumption did not differ between the three experimental diets, although differences in protein, fat and NFE intake were observed according to each diet composition (*P* < 0·05).

**Table 1.** Nutrient intake of dogs, cats and ferrets fed extruded diets with different macronutrient compositions

		Intake (g/kg BW per d)*				
Item	Diet	Dogs (*n* 6)	Cats (*n* 6)	Ferrets (*n* 6)	Mean	sem†
	HC	17·6^B^	9·9^A^	29·3^C^	18·2	5·6
	MC	17·7^A^	10·8^A^	31·1^B^	19·0	6·0
DM	LC	12·3^A^	14·4^A^	31·3^B^	19·3	6·0
	Mean	15·5	12·1	30·7		
	sem	1·8	1·4	0·7		
Protein	HC	5·4^aA^	4·4^aA^	8·9^aB^	5·5	1·4
	MC	7·3^aB^	4·3^aA^	12·8^bC^	7·8	2·5
	LC	5·6^aA^	6·5^bA^	14·3^bB^	8·8	2·8
	Mean	6·0	5·0	12·6		
	sem	0·6	0·7	1·6		
Fat	HC	1·3^aAB^	0·7^aA^	2·2^aB^	1·4	0·4
	MC	1·8^aA^	1·1^aA^	3·2^aB^	1·9	0·6
	LC	2·8^bA^	3·3^bA^	7·2^bB^	4·4	1·4
	Mean	2·1	2·0	4·9		
	sem	0·4	0·8	1·5		
Nitrogen-free extract	HC	9·5^bB^	5·3^bA^	15·8^bC^	9·8	3·1
	MC	6·5^abA^	4·0^bA^	11·5^abB^	7·0	2·2
	LC	2·0^bA^	2·7^aA^	5·8^aB^	8·8	2·8
	Mean	7·1	5·3	14·0		
	sem	1·2	0·7	1·3		

HC, high carbohydrate; MC, medium carbohydrate; LC, low carbohydrate.

^a,b^ Within a column, means not sharing a common superscript differ (*P* < 0·05). Comparison valid inside a nutrient.

^A,B,C^ Within a row, means not sharing a common superscript differ (*P* < 0·05).

*Dogs and cats were fed calculated amounts of food and ferrets were fed *ad libitum*.

†Standard error of mean, *n* 6 animals per diet.

The digestibility of DM and NFE presented a diet × species interaction (*P* < 0·05; Table 2). Dogs and cats presented similar DM and NFE digestibility for the three diets, but ferrets presented lower digestibility values (*P* < 0·01). In the comparison of diets within each species, dogs presented higher DM digestibility for the HC diet (*P* < 0·05), but ferrets presented higher DM digestibility for the LC diet (*P* < 0·05), and cats showed lower apparent total tract digestibility (ATTD) of DM for the MC diet (*P* < 0·05). For protein and fat digestibility, however, only a species effect was observed: the digestibility of protein was higher for dogs than for both cats and ferrets and higher for cats than for ferrets (*P* < 0·05). For fat, dogs and ferrets presented similar digestibility, and values for both species were higher than those for cats (*P* < 0·05).

**Table 2.** Coefficients of total tract apparent digestibility of nutrients observed on dogs, cats and ferrets fed extruded diets with different macronutrient compositions

		Digestibility (%)*				
Item	Diet	Dogs (*n* 6)	Cats (*n* 6)	Ferrets (*n* 6)	Mean	sem†
	HC	83·3^bB^	79·0^bB^	68·9^aA^	76·4	4·3
	MC	78·3^aB^	74·3^aB^	67·5^aA^	74·1	3·2
DM	LC	81·4^abB^	80·3^bB^	74·3^bA^	78·7	2·2
	Mean	81·1	78·0	70·8		
	sem	1·5	1·8	2·1		
Protein	HC	86·2	80·5	74·0	79·7^a^	3·5
	MC	87·0	83·38	72·1	82·0^ab^	4·5
	LC	86·9	85·4	79·5	83·9^b^	2·3
	Mean	86·8^C^	83·2^B^	75·8^A^		
	sem	0·2	1·4	2·2		
Fat	HC	87·5	83·1	85·8	85·4^a^	1·3
	MC	88·4	85·3	89·4	87·0^b^	1·3
	LC	94·5	91·5	94. 0	93·5^c^	1·0
	Mean	90·6^B^	86·6^A^	90·0^B^		
	sem	2·2	2·5	2·6		
Nitrogen-free extract	HC	90·0^bB^	86·3^aB^	70·4^bA^	81·3	6·0
	MC	82·0^aB^	79·6^aAB^	71·5^abA^	78·5	3·2
	LC	82·1^aB^	85·6^aB^	63·4^aA^	77·0	6·9
	Mean	84·4	83·9	67·89		
	sem	2·6	2·1	2·5		

HC, high carbohydrate; MC, medium carbohydrate; LC, low carbohydrate.

^a,b^ Within a column, means not sharing a common superscript differ (*P* < 0·05). Comparison valid inside a nutrient.

^A,B,C^ Within a row, means not sharing a common superscript differ (*P* < 0·05).

*Dogs and cats were fed calculated amounts of food and ferrets were fed *ad libitum*.

†Standard error of mean, *n* 6 animals per diet.

## Discussion

The results suggest that extruded kibble diets of different macronutrient compositions, largely varying in NFE content, are eaten and digested by ferrets, which produce adequately formed faeces. However, ferrets presented limited DM, NFE and protein ATTD compared with dogs and cats. Ferrets can digest fat better than cats, but not better than dogs. Conclusions regarding food intake from the present study are limited because it was assessed over a very short time, restricted to only 15 d of food intake. Even so, it is remarkable that ferrets ate approximately twice as much food as dogs and cats per kg BW and low nutrient digestibility could be one of the possible reasons.

A study about feeding habits of ferrets and feral cats in New Zealand^(^^10^^)^ observed that both species have similar dietary habits, consuming the same types of preys (lagomorphs, birds and rodents). This may suggest a similar digestive physiology, but the lower DM digestibility and the higher fat digestibility for ferrets as compared with cats, limits the value of extrapolations between the two species. Other authors^(^^11^^)^ had already shown lower CP (70·1 ± 5·4 *v*. 75·9 ± 5·8) and higher crude fat (95·6 ± 1·5 *v*. 89·4 ± 5·3) digestibility for ferrets as compared with cats when fed three different diets (‘normal diet’ (37·6 % NFE, 33·7 % CP, 20·4 % fat); ‘light diet’ (52·2 % NFE, 31·6 % CP, 10·7 % fat); and ‘veterinary diet’ (47·2 % NFE, 38·7 % CP, 9·6 % fat)), results that are in agreement with those of the present study.

Diets formulated for cats have been recommended for ferrets, but this practice may be questionable since a lower protein digestibility for ferrets may increase the requirement for this nutrient in practical diets and that the lower NFE and higher fat digestibility for ferrets suggests different use of dietary energy sources. It is possible that ferrets are more adapted to a higher fat intake, making fat an energy source that is preferred over carbohydrates. The reduced NFE and protein digestibility exhibited by ferrets could be related to the short gastrointestinal tract and intestinal transit time^(^^1^^)^, but future studies are needed to better understand this. Furthermore, true digestibility was not measured, only ATTD was measured, and other factors such as microbial interference with nutrient faecal excretion may have influenced the results.

No other studies comparing the digestive efficiency of dogs and ferrets were found. Comparing the digestibility of diets fed to a *Canidae*, the blue fox (*A. lapogus*), with that of a *Mustelidae*, the mink^(^^3^^)^, others reported higher nitrogen, amino acid and fat digestibility for the blue fox, corroborating the results found for dogs in the present study.

It is reported that dogs have higher nutrient digestibility than cats^(^^6^^)^. When dogs and cats were compared, dogs had a higher digestibility of DM, organic matter, NFE and fat when fed wet, semi-purified experimental diets or dry extruded diets^(^^5^^)^. Lower digestibility of protein and fat for cats was verified in the present study, but this was not found for DM and NFE. Explanations for these differences could be related to the sources of carbohydrates used in the two studies, the quality of food processing and starch cooking or the amount or type of fibre in the diets. The NFE uses the CF analysis for computations, an analysis that presents several important problems and incomplete fibre quantification. The fibre not quantified in the CF analysis is incorrectly computed as NFE, but in fact has low digestibility and energy value^(^^12^^)^. This methodological limitation of the available studies limits comprehension of the differences in carbohydrate digestibility among dogs, cats and ferrets.

## Conclusion

Ferrets fed dry kibble diets presented lower DM, NFE and protein digestibility than dogs and cats. However, ferrets present fat digestibility similar to that of dogs and higher than that of cats. Therefore, to formulate practical diets for ferrets it is important to consider the quality and amount of protein, and the use of fat as an energy source.
